# Personality traits and medical specialty preference among medical students and graduates: a scoping review

**DOI:** 10.3325/cmj.2025.66.321

**Published:** 2025-10

**Authors:** Antonia Peroš, Nensi Bralić, Ivan Buljan

**Affiliations:** 1 Career Management Office, University of Split, Split, Croatia; 2 Department of Research in Biomedicine and Health, University of Split School of Medicine, Split, Croatia; 3 Department of Psychology, Faculty of Humanities and Social Sciences, University of Split, Split, Croatia

## Abstract

**Aim:**

To review the available research on the association between personality traits and specialty choice.

**Methods:**

A systematic search of MEDLINE, ERIC, Scopus, PsycINFO, and Web of Science was performed in June 2022 and updated on August 18, 2025. Studies were eligible if they examined the association between personality traits and specialty choice among medical students and graduates using validated psychological tests. The protocol was registered at the Open Science Framework (https://osf.io/su6md). Screening, eligibility assessment, and data extraction were performed independently by two reviewers.

**Results:**

A total of 9212 articles were retrieved, of which 61 met the inclusion criteria. Considerable heterogeneity in study design, instruments used, and outcomes assessed precluded quantitative synthesis. Most studies indicated associations between personality traits and specialty choice, although findings were inconsistent. Surgery was the most frequently assessed specialty, with several studies reporting higher impulsivity, higher extraversion, higher openness to new experiences, and a lower desire to work with people among students choosing surgery. Psychiatry, internal medicine, and family medicine were also frequently examined, but associations varied across studies. Methodological limitations were common, including reliance on cross-sectional designs, inconsistent measurement tools, and underreporting of psychometric properties.

**Conclusion:**

Evidence suggests a relationship between personality traits and specialty choice; however, this evidence remains weak due to methodological shortcomings. Future studies should apply standardized measurements for both personality traits and specialty choice, with larger and more diverse samples.

The choice of specialty is an important turning point in the life of every medical student. To most of physicians, becoming a specialist represents not only an important career decision but also, from a social perspective, an enhancement in their quality of life and the attainability of new job opportunities (1).

Preferences and factors influencing medical career choices play a crucial role in the dynamic, complex, and multifactorial process of deciding on a medical career path (2-4). Factors influencing specialty preference are grouped into five categories: characteristics of the medical school, such as curriculum design; student characteristics, including age and personality; personal values; career goals, including income, status, and work-life balance; and perceptions of specialty attributes shaped by curricular or extracurricular experiences (3). Specialty preference is therefore not a simple matter of student decision but a complex interplay of student preferences, program priorities, and competition for available positions (2).

Many student characteristics are tied to key psychological traits. Although some studies found associations between personality and specialty preference (1,5-7), the results were conflicting, even after accounting for setting-specific variables (6). Some studies showed that specialists in surgical fields such as general surgery, gynecology, or traumatology tend to be more extroverted and organized (1). Others indicated that specialists outside core surgical specialties, including pediatrics, ophthalmology, dermatology, orthopedics, psychiatry, and general practice, tend to be more introverted, anxious, and emotionally connected to patients (1). Several authors suggested that the relationship between personality and specialty preference remains unclear (6,8-11). An additional obstacle to the generalizability of these findings is the use of different personality measures and different categorizations of specialties in different available studies.

The existing systematic reviews on the relationship between personality traits and specialty preference among medical students and graduates do not provide sufficient evidence. They explored different outcomes, did not focus in detail on personality traits, and did not consider the complexity of the concept (3,8). Moreover, most reviews examined personality traits without assessing the validity of personality tests (8-11). To clarify the relationship between specialty preference and personality traits, researchers need a rigorous and systematic approach. Such an approach would provide more reliable knowledge and a better understanding of the issue, which is essential for offering effective medical career consultation during and after medical education. This, in turn, would support students in choosing their specialty and improve the person-job fit among physicians (1,4,6,12). The aim of this scoping review is to provide an overview of the available research evidence regarding the association between personality traits and specialty preferences among medical students and graduates.

## METHODS

Considering the broad aims of the study and its intention to identify gaps in knowledge, we chose a scoping review as a valuable tool for evidence synthesis, following the Joanna Briggs Institute (JBI) methodology (13). The review adhered to the Preferred Reporting Items for Systematic Reviews and Meta-Analysis (PRISMA-SR) guideline (14) (Supplemental Material 1(Supplementary Material 1)), and we registered the protocol at the Open Science Framework (https://osf.io/su6md; DOI: 10.17605/OSF.IO/WR23B) on June 24, 2022.

### Eligibility criteria

We structured the inclusion criteria using the PICO framework (population, intervention, comparator, and outcome) with the addition of study design (15).

Population: Medical students and graduates.

Intervention: Association between personality traits and specialty preference.

Comparator: Not applicable.

Outcome: Studies on specialty preference and personality traits measured with validated psychological tests. Psychological tests were defined as “any psychometrically derived measurement instrument that assesses psychological constructs by obtaining a structured sample of behavior in a specified domain, quantified, scored, interpreted, and synthesized using a standardized process for evaluative conclusions or recommendations” (16).

Study design: Quantitative studies, including descriptive, correlational, causal-comparative/quasi-experimental, and experimental designs. We excluded qualitative studies because they did not assess personality traits with scientifically validated psychological tests.

Other: No language restrictions were applied.

### Information sources

We identified relevant studies through systematic searches of the following bibliographic databases, with no time restrictions: MEDLINE (Medical Literature Analysis and Retrieval System Online) (June 6, 2022), ERIC (Education Resources Information Center) (June 2, 2022), Scopus (June 6, 2022), PsycINFO (June 2, 2022), and Web of Science (June 6, 2022). The initial systematic search was conducted in June 2022 using a comprehensive strategy developed specifically to address the research question. This strategy combined controlled vocabulary (eg, MeSH terms) and free-text keywords, adapted to the indexing system of each database, and was verified by a professional librarian (Supplemental Material 2(Supplementary Material 2)). The core search string included three main concepts: medical students (eg, “medic* students,” “medic* graduates,” “undergraduates in medicine”), personality (eg, “personalit*,” “personality trait*,” “personality assessment*”), and career preference (eg, “specialt*,” “specialty,” “career preference*”). We linked these concepts with Boolean operators (AND/OR) and applied truncation where appropriate to maximize sensitivity and specificity. To ensure inclusion of the most recent literature, we performed an updated search across all databases on August 18, 2025 using the same strategy.

### Selection of sources of evidence

We exported the search results to EndNote for organization. Two reviewers (AP, NB) independently screened the studies in two steps: first by reviewing the titles and abstracts, then by analyzing full texts and screening the reference lists of included systematic reviews. They resolved disagreements through consensus or consultation with a third reviewer (IB). We reported excluded studies and reasons for exclusion in Supplemental Material 3(Supplementary Mateiral 3), available at *https://osf.io/wr23b/.*

### Data extraction

The third step was data extraction. Two reviewers (AP, NB) developed a data-charting form in Microsoft Excel to guide the process. They pilot-tested the form on a representative sample of 10 studies. Both reviewers independently extracted data, discussed results, and continuously updated the form throughout.

### Data items

We extracted the following data: publication type, preregistration status, country of origin, study aims, population, level of education, sample type, sample size, response rate, setting, study design, data collection period, outcomes measured, instruments used, reliability measurement, reliability coefficient value and size (if applicable), type of validation, specialty preference assessment, key findings, statistical analyses, coefficients between traits and specialty preference (if applicable), suggestions for future research, study limitations, ethics approval (including the granting body), and whether data were shared.

### Critical appraisal of individual sources of evidence

We assessed the methodological quality of individual studies using the Joanna Briggs Critical Appraisal Tool (17). For longitudinal studies, we used the JBI Critical Appraisal Checklist for Cohort Studies, and for cross-sectional studies, we used the JBI Critical Appraisal Checklist for Analytical Cross-Sectional Studies (18). In both tools, each criterion could be scored as met (yes), unmet (no), unclear, or not applicable. Two reviewers (AP, IB) independently assessed the studies.

### Synthesis of results

Because of significant variations in populations, research methodologies, personality traits assessed, and statistical approaches, we analyzed and presented the data descriptively and separately for longitudinal and cross-sectional studies.

### Data analysis

We analyzed and summarized the data descriptively. Study characteristics were tabulated in Supplemental Material 4(Supplementary Material 4) and summarized narratively in the text. We presented descriptive data as frequencies, with medians for sample sizes and percentages for categorical variables calculated using Microsoft Excel (2016; Microsoft, Redmond, WA, USA).

## RESULTS

**Figure 1:**
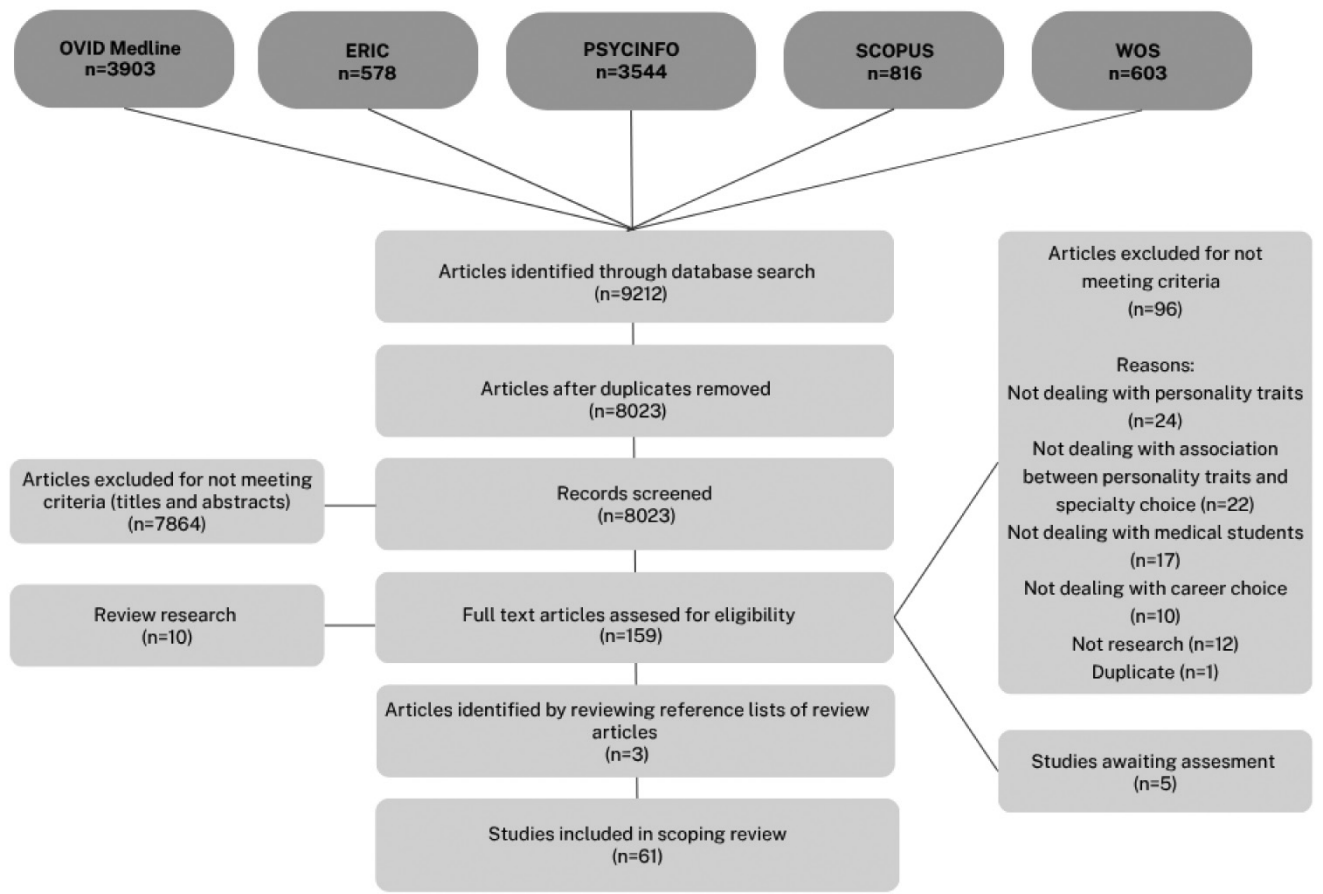
PRISMA flow diagram for the scoping review process.

A total of 9212 articles were initially retrieved. After removing duplicates, 8023 remained for screening at the title and abstract level. Most were excluded at this stage because they did not focus on personality traits, were not associated with specialty preference, or did not involve medical students. Of the screened records, 159 articles were selected for full-text assessment, and 58 were included in the scoping review. The reasons for exclusion in this step were not meeting the eligibility criteria (n = 96) and insufficient information or lack of full-text availability (n = 5). Supplemental Material 3(Supplementary Material 3) (Sheet Analysis 1) lists the full references of the studies excluded at the full-text screening stage (n = 159), along with the main reasons for exclusion. An additional three studies were identified through reference list screening, and the final number of studies included in the review was 61. All steps of the selection process are summarized in Figure 1.

### CROSS-SECTIONAL STUDIES

Forty-seven cross-sectional studies were conducted between 1955 and 2024, all published as journal articles. Most studies included undergraduate medical students, with the largest proportion from the USA (n = 16). The median participant age was 23.7 years (interquartile range [IQR] 22-24.3). Most studies relied on convenience samples (n = 39). The median sample size was 610 (IQR 169-710), with a median response rate of 76% (IQR 60-95). In most cases, the study setting was not described. Four studies were conducted onsite rather than online, which is consistent with the years of publication. Most studies assessed multiple personality traits (Table 1).

**Table 1:** Personality measures used in the analyzed cross-sectional studies

Test	No.	References
The Neuroticism-Extraversion-Openness Personality Inventory	5	(19-23)
Myers-Briggs Type Indicator	4	(24-27)
Cattell Test 16 Personality Factors	3	(28-30)
The Machiavellism test IV	1	(31)
The Machiavellism test V	1	(32)
The California Authoritarian (F) scale	2	(31,33)
The Maudsley Personality Inventory	2	(34,35)
The Rokeach Dogmatism Scale	3	(36-38)
Edwards Personal Preference Schedule	2	(39,40)
Intolerance of Ambiguity Scale developed by Budner	1	(41)
Rotter's Internal-External Locus of Control (I-E Scale)	2	(37,42)
The State-Trait Anxiety Inventory Form X-2	2	(37,43)
Eysenck Personality Questionnaire	2	(44,45)
German Extended Personal Attributes Questionnaire	1	(46)
Mini-International Personality Item Pool (IPIP)	2	(47,48)
The Zuckerman-Kuhlman Personality Questionnaire-Medium Form	2	(49,50)
Cloninger’s Temperament and Character Inventory-140	2	(43,51)
Big Five Inventory	2	(4,52)
International Personality Item Pool Big-Five Factor Marker	3	(53-55)
Psychopathic Personality Traits Inventory	1	(56)
Ten-Item Personality Inventory	1	(57)
The HEXACO Personality Inventory – Revised (self-report form)	1	(1)
Croatian short version of the IPIP Big-Five questionnaire	1	(12)
Big Five Inventory-2-S 30-item Scale	1	(58)
German short version of the Big Five Inventory	1	(59)
NERIS Type Explorer	1	(60)

The reliability of these tests was not measured in most studies (n = 44). When reported, reliability and validation were usually cited from previous research, with only a few studies providing direct data (4,40,47,51). Specialty preference was mainly assessed with questionnaires, either by grouping specialties into categories (n = 15) (7,12,19,28,30,31,33,36,41,47,49,55,58,59) or reducing them to binary outcomes (n = 11) (1,4,23,25,29,43,50,53,56,57).

The most common statistical analyses were ANOVA (n = 13) and regression analysis (n = 10). Almost half (n = 22) of the studies did not report ethical approval. None shared their data, and only a small number indicated they would share data on request (33,45,48,52,53,59,60).

**Key findings**. Eight studies reported no significant association between personality traits and specialty preference (12,23,43,44,46,52,61,62). The most frequently assessed specialties were surgery (n = 25) and psychiatry (n = 20). Other results on the association between certain personality traits and the most frequent specialty preferences are presented in Table 2.

**Table 2:** Findings in cross-sectional studies assessing specialties

Finding	No.	References
Surgery (n = 25)		
no association found	3	(30,40,49)
higher impulsivity	4	(1,25,31,63)
lower neuroticism	5	(1,25,28,54,62)
higer extraversion	4	(1,55,58,62,64)
higer openness	3	(54,55,62)
lower conscientiousness	2	(58,64)
higer conscientiousness	1	(54,64)
lower harm avoidance, fear of uncertainty, shyness and sentimentality	1	(32)
lower agreeableness	1	(59)
thinking trait	1	(60)
Psychiatry (n = 20)		
no association found	3	(40,44,52)
higher openness	3	(20,22,49)
higher neuroticism	2	(48,49)
higher extraversion	3	(49)
highly oriented to the theoretical and abstract, altruistic, preference to analyze the behavior of self and others	1	(37)
intuitive (men)	1	(19)
emotionally immature, sensitive and dependent, impulsive, unstable and disorderly	1	(51)
higher anxiety and higher anger, tension and confusion	1	(26)
sensible and imaginative	1	(56)
high tolerance for ambiguity	2	(43,56)
introversion, intuition, judging as significant predictors	1	(12)
higher authoritarianism, lower death anxiety	1	(29)
intuitive and prospective traits	1	(60)
Internal medicine (n = 10)		
no association found	2	(30,56)
lower authoritarianism	1	(4)
higher authoritarianism	1	(29)
lower openness	1	(29)
higher conscientiousness	1	(29)
lower in avoidance, fear of uncertainty, shyness, and sentimentality	1	(32)
introverted-sensing-thinking-perceptive	1	(46)
intuitive, judging style (men only)	1	(19)
lower extraversion	1	(59)
Anesthesiology (n = 4)		
higher conscientiousness and neuroticism	2	(65,66)
lower openness	1	(65)
lower neuroticism and openness	1	(59)
Gynecology (n = 6)		
introverted-sensing-thinking-perceptive personality type	1	(46)
higher conscientiousness	1	(65)
lower openness	1	(65)
higher tolerance for ambiguity	1	(56)
sensing	1	(19)
neuroticism	1	(59)
Radiology (n = 1)		
higher neuroticism and lower openness	1	(65)
Pediatrics (n = 4)		
introverted-sensing-thinking-perceptive personality type	1	(46)
lower openness and higher conscientiousness	1	(65)
higher anxiety	1	(29)
agreeableness	1	(59)
Family medicine (n = 8)		
lower harm avoidance	1	(42)
higher reward dependence	1	(42)
lower neuroticism	1	(49)
higher agreeableness	1	(30,67)
higher authoritarianism	1	(4)
lower in openness	1	(65)
higher conscientiousness	1	(65)
introverted, observant, and judging	1	(60)

**Critical appraisal of individual sources of evidence**. Methodological quality issues were present in most cross-sectional studies (n = 37, 78.7%), with deficiencies in at least one item of the Critical Appraisal Checklist for analytical cross-sectional studies (Supplemental Material 5(Supplementary Material 5)).

### LONGITUDINAL STUDIES

Fourteen longitudinal studies were conducted between 1964 and 2024, all published as journal articles. Most included medical students from the USA (n = 7), and many focused on graduates (n = 7). The median participant age was 22.4 years (IQR 22.2-23.8). The median sample size was 302 (IQR 199-362), with a median response rate of 65% (IQR 49-76). Settings were usually not described, although three studies were conducted online (68-70). The personality measures used in longitudinal studies are shown in Table 3.

**Table 3:** Personality measures used in the analyzed longitudinal studies (N = 14)

Measure	No.	References
Myers-Briggs Type Indicator	2	(24-27,71,72)
Edwards Personal Preference Schedule	2	(73,74)
Cattell 16 Personality Factor Questionnaire	2	(75,76)
Eysenck Personality Questionnaire	1	(77)
German Extended Personal Attributes Questionnaire	1	(77)
The Depressive Experiences Questionnaire	1	(78)
The Career Occupational Preference System Inventory	1	(9)
Strong Interest Inventory Personal Style Scales	1	(9)
Temperament and Character Inventory-Revised	1	(49,50,68)
The Neuroticism-Extraversion-Openness Personality Inventory (Five-Factor)	1	(69)
Tolerance for Ambiguity Scale	1	(79)
Ambiguity Aversion in Medicine Scale	1	(79)
Big Five Inventory-10	1	(79)
German short version of the Big Five Inventory	1	(70)

Reliability was not measured directly in these studies but cited from prior research.

Specialty preference was most often assessed with questionnaires (n = 8) (9,68-71,74,76,79), sometimes grouping specialties into categories (n = 4) (70,73,77,78). Two studies used standardized questionnaires from the American National Resident Matching Program (72,75,77).

The most common statistical analyses were ANOVA (n = 2), MANOVA (n = 2), and logistic regression (n = 3). Only four studies reported ethical approval (n = 4) (68,70,74,75), and none shared their data.

**Key findings**. Surgery was the most frequently reported specialty. Over half of the studies (n = 8) reported a connection between surgical specialty preference and personality traits (70-72,74,75,77-79). One longitudinal study reported no significant association between personality traits and specialty preference (73).

The findings from longitudinal studies on the relationship between personality traits and commonly preferred specialties are presented in Table 4. Because of study heterogeneity, we reported the results separately for the most frequently assessed specialties. Surgery (n = 7) and psychiatry (n = 4) were the most frequently reported preferences.

**Table 4:** Findings in longitudinal studies assessing specialty prediction

Finding	No.	References
Surgery (N = 7)		
lower desire to work with people	2 (28.6)	(9,74)
higher neuroticism	2	(71,79)
low tolerance for risk and ambiguity	1	(79)
moderate tolerance for complexity	1	(79)
high in masculine traits	3	(79) (75) (77)
high self-efficacy	1	(78)
thinking types with high scores on extraversion	1	(72)
Psychiatry (N = 4)		
high levels of self-criticism	1	(78)
intuitive-feeling-perceiving types	1	(9)
lower extraversion	1	(68)
introversion	1	(79)
Anesthesiology (N = 3)		
lower desire to work with people	1	(74)
low self-criticism	1	(78)
no association found	1	(9)
Gynecology (N = 3)		
higher desire to work with people	1	(74)
sensing, thinking, and judging personality types	1	(77)
higher neuroticism	1	(68)
low aggression	1	(68)
Clinical pathology (N = 1)		
high harm avoidance	1	(77)
Radiology (N = 1)		
low harm avoidance	1	(77)
Pediatrics (N = 2)		
high stability	1	(9)
external locus of control, scored high on warmth and helpfulness (femininity)	1	(9)
high feminine traits of warmth and helpfulness	1	(9)
high masculine traits of competitiveness, confidence, and independence	1	(9)
high extraversion	1	(79)
Family medicine prediction (N = 5)		
low self-criticism	1	(78)
lower need to exercise leadership	1	(76)
sensing, thinking, and judging personality types	1	(9)
feeling types, higher introversion	1	(72)
higher agreeableness and neuroticism	1	(70)

**Critical appraisal of individual sources of evidence**. Almost half of the longitudinal studies (n = 6, 46.1%) had methodological issues, particularly related to confounding factors (Supplemental Material 5(Supplementary Material 5)).

## DISCUSSION

This scoping review included 61 studies spanning nearly seven decades, providing one of the most comprehensive mappings of the relationship between personality traits and medical specialty preference among students and graduates. Many studies reported associations between personality traits and specialty preference; however, the findings were inconsistent and often contradictory. As a result, no systematic pattern emerged that would allow reliable prediction of specialty preference based solely on personality traits.

The included studies were highly heterogeneous in terms of populations, methodologies, instruments, traits assessed, and statistical approaches. Methodological limitations were common, which increased the risk of bias. Most studies used cross-sectional designs, which limited conclusions about the stability of specialty preferences over time. Although longitudinal studies were fewer in number, they provided some evidence that personality traits may be related to specialty preference, but the associations were not consistent across traits or specialties. This dynamic pattern underscores the need for more longitudinal research to clarify the stability and evolution of specialty preferences.

One of the major challenges lies in the diversity of personality measures. While instruments such as the Neuroticism-Extraversion-Openness Personality Inventory, the Big Five Inventory, the Eysenck Personality Questionnaire, and the Mini- International Personality Item Pool were frequently used, many other tools were applied sporadically. This variety complicates comparison across studies and weakens the strength of cumulative evidence. Similarly, a recent study identified the proliferation of psychological measures as a severe barrier to cumulative science (80). In addition, reliability and validity of the measures were often underreported, with many studies citing psychometric properties from previous research rather than presenting direct evidence from their own data. These shortcomings highlight the need not only for more consistent use of validated instruments but also for rigorous and transparent reporting of psychometric properties. Evidence for scale validity and reliability in social and personality research is severely underreported, a problem also recognized in a recent study (81). Such underreporting contravenes the American Psychological Association’s reporting guidelines, which emphasize the need to provide reliability and validity evidence for self-report questionnaires (82).

Assessment of specialty preference also lacked standardization (75,77). Most questionnaires grouped specialties into broad or binary categories, often with unequal representation.

Another problem related to specialty choice was an unequal number of specialties represented in studies. Surgery, for example, was disproportionately studied, which reflected both research trends and global shortages in surgical specialties. Both European and American national data are putting surgery, psychiatry, and family medicine on top of the specialties in demand (83,84). Our findings showed that surgery was frequently associated with impulsivity (1,31,63,85), higher extraversion (25,29,55,58,62,64), higher openness to new experiences (54,55,62), and a lower desire to work with people (74,78). Several longitudinal studies found that a set of masculine traits can predict surgery as a specialty choice (75,77,79). However, results on conscientiousness (20,54,58,64) and neuroticism (1,25,54,62,79,86) were divided.

These mixed findings are consistent with previous reviews and empirical studies. For example, Borges (87) observed similar but inconsistent trends in his early synthesis of personality and specialty choice. Nawaiseh et al (4) found that while personality traits do influence specialty preference – identifying links between certain traits and specialties such as surgery, psychiatry, pediatrics, and internal medicine – the patterns remain complex and variable. Similarly, most recently, Tobiaszewska et al (60) demonstrated gender-based differences in how personality types align with specialty preferences among over 2000 medical students, illustrating further nuance and heterogeneity in the associations.

Besides the issues related to outcome assessment, another challenge in determining the association between personality traits and specialty preference among medical students and graduates was the predominance of cross-sectional over longitudinal designs. Cross-sectional studies were linked to several commonly reported limitations (23,25,27,30,31,48,58,59,64). This type of design cannot establish whether specialty preference is fixed or subject to change during medical training, a limitation also noted in several studies (7,26,48,55,64,66,74,85,88). This question was addressed multiple times in the longitudinal studies included in the analysis. One study confirmed the stability of specialty preference over time in medical students (69).

The gaps in knowledge identified in this scoping review primarily concern outcome measurement. Future research should address these gaps by applying standardized instruments for both personality traits and specialty preference. Another key recommendation concerns sample characteristics: studies should include larger sample sizes, with participants drawn from different generations, universities, and countries.

It is also important to consider additional factors such as life circumstances and personal decisions (52,60,75,78), sex differences in work style preferences (9,60), specific national factors (4), and socioeconomic changes in Europe (39). Levaillant et al (8) also highlighted how lifestyle and work-life balance play critical roles in shaping preferences, sometimes outweighing personality effects.

Future studies should also investigate how personality traits influence both the selection and the practice of different specialties (68,72,73,79). Moreover, research should examine how personality traits contribute not only to career choice but also to physician satisfaction, satisfaction with specialty preference, and retention (75). Some studies suggest that career preference is a complex issue best addressed through large-scale national studies that enable multivariate analyses (55,77,78).

The findings highlight the potential value of integrating personality assessment into medical career guidance during and after medical education, in order to improve person-job fit and physician satisfaction (1,12,59). Such evidence can also be considered when organizing psychological intervention programs targeted at educating medical students.

Personality testing for selection into medicine is an area of growing interest (89). Healthcare institutions may benefit from developing selection criteria for specialty training candidates, with added advantages for universities and health care institutions given the relatively low cost of psychological testing.

This study has several limitations. Although we conducted a broad and sensitive search of relevant databases, one limitation is the exclusion of gray literature, which was initially planned. For this reason, some relevant studies may have been omitted. However, since gray literature does not reliably add value (90), we considered this exclusion unlikely to affect the quality of our findings. Another limitation is that we restricted our search to specialty preferences. Such preferences may not always represent fixed or stable career decisions, but they can serve as a starting point in medical career development.

In conclusion, evidence suggests a relationship between personality traits and specialty preference; however, the overall evidence remains weak due to the lack of standardized measurements. Future research should address these gaps by using validated instruments for both personality traits and specialty preference, applied to larger and more diverse samples. This relationship should be further explored for advancing medical career counseling, improving specialty selection, and strengthening the education of future physicians.
