# Supplementary material for: Personality traits and medical specialty preference among medical students and graduates: a scoping review
Source: Croat Med J. 2025 Oct;66(5):321–33. doi: 10.3325/cmj.2025.66.321 (PMC12631570; doi:10.3325/cmj.2025.66.321)
Supplement: Supplementary Material 2 [file CroatMedJ_66_s002.pdf]

Supplemental Material 2

Search strategy OVID Medline

1 exp Students/ (152970)

2 exp Students, Medical/ (40076)

3 Education, Medical, Graduate/ (32119)

4 Education, Medical, Undergraduate/ (26247)

5 (medical adj3 (student* or graduate* or undergraduate*)).tw. (65233)

6 1 or 2 or 3 or 4 or 5 (224592)

7 exp Personality/ (381294)

8 MMPI/ (6970)

9 Type D Personality/ or Personality Inventory/ or Personality Tests/ or Type B Personality/ or Personality Assessment/ or Cattell Personality Factor Questionnaire/ or Type A Personality/ or Personality Development/ (60104)

10 MMPI.tw. (5156)

11 type d personality. tw. (647)

12 Type B Personality.tw. (16)

13 Type A Personality.tw. (223)

14 (personality adj3 (trait* or measure* or construct* or factor* or inventor* or assesment* or test*)).tw. (28853)

15 Individuality/ (15627)

16 individuality.tw. (3247)

17 7 or 8 or 9 or 10 or 11 or 12 or 13 or 14 or 15 or 16 (427345)

18 Specialization/ (25278)

19 specialization*.tw. (22343)

20 Career Choice/ (24880)

21 career*.tw. (48334)

22 "Fellowships and Scholarships"/ (8762)

23 fellows*.tw. (19793)

24 "Internship and Residency"/ (56710)

25 internship*.tw. (4086)

26 residency.tw. (31947)

27 (career adj3 (choice* or preference*)).tw. (3504)

28 18 or 19 or 20 or 21 or 22 or 23 or 24 or 25 or 26 or 27 (188647)

29 6 and 17 and 28 (3259)

+ 644 (18 August 2025)

Search strategy ERIC

1 exp Students/ (386171)

2 exp Medical Students/ (4793)

3 exp Graduate Medical Education/ or exp Medical Education/ or exp Graduates/ (32020)

4 (medical adj3 (student* or graduate* or undergraduate)).tw. (7524)

5 exp Personality Traits/ or exp Personality Assessment/ or exp Personality Measures/ or exp Personality/ (25110)

6 (personality adj3 (trait* or measure* or construct* or factor* or inventor* or assessment* or test*)).tw. (17756)

7 5 or 6 (26999)

8 exp Specialization/ (2318)

9 specialization*.tw. (5072)

10 specialty.tw. (2357)

11 exp Careers/ (4394)

12 (career adj3 (choice* or preference*)).tw. (14210)

13 8 or 9 or 10 or 11 or 12 (24529)

14 1 or 2 or 3 or 4 (406769)

15 7 and 13 and 14 (521)

+57 (18 August 2025)

Search strategy PSYCINFO

(medic* students or students or medic* graduates or medic* undergraduates or students in medicine) AND (personalit* or personality trait* or personality measure* or personality construct* or personality factor* or personality inventor* or personality assessment* or personality test*) AND (specialt* or specialization or career* or career choice* or career preference*)

Search Results: 3,544

Search strategy SCOPUS

(TITLE-ABS-KEY (medic* AND students) OR TITLE-ABS-KEY (medic* AND graduat*) OR TITLE-ABS-KEY (medic* AND undergraduat*) AND TITLE-ABS-KEY (personalit*) OR TITLE-ABS-KEY (personality AND trait*) OR TITLE-ABS-KEY (personality AND measure*) OR TITLE-ABS-KEY (personality AND assessment*) OR TITLE-ABS-KEY (personality AND construct*) OR TITLE-ABS-KEY (personality AND factor) OR TITLE-ABS-KEY (personality AND inventor*) OR TITLE-ABS-KEY (personality AND test*) AND TITLE-ABS-KEY (specialt*) OR TITLE-ABS-KEY (specialization) OR TITLE-ABS-KEY (career*) OR TITLE-ABS-KEY (career AND choice*) OR TITLE-ABS-KEY (career AND preference*))

Search Results: 816

Search strategy WOS

(ALL=(medic* students)) OR ALL=(medic* graduate*)) OR ALL=(medic* undergraduat*)

((((((ALL=(Personalit*)) OR ALL=(personality trait*)) OR ALL=(personality measure*)) OR ALL=(personality construct*)) OR ALL=(personality factor*)) OR ALL=(Personality inventor*)) OR ALL=(personality assessment*)) OR ALL=(personality test*)

((((ALL=(specialt*)) OR ALL=(specialization*)) OR ALL=(career*)) OR ALL=(career choice*)) OR

ALL=(career preference*)

Search Results: 603
