# Supplementary material for: Personality traits and medical specialty preference among medical students and graduates: a scoping review
Source: Croat Med J. 2025 Oct;66(5):321–33. doi: 10.3325/cmj.2025.66.321 (PMC12631570; doi:10.3325/cmj.2025.66.321)
Supplement: Supplementary Material 4 [file CroatMedJ_66_s004.pdf]

Supplemental Material 4

Table 1. Overview of characteristics of studies about the association between personality traits and specialty preferences among medical students and graduates.

Title	Year published	Population of interest	Sample size	Study design	Outcomes assessed_ personality	Outcomes assessed_ medical	Findings recoded
Personality Traits and Potential Career Choices Among Medical Students at Sultan Qaboos University: A Cross-Sectional Study	2024	Medical students	234	Cross-sectional	Multiple traits	Preffered specialty	These results indicate that the medical students who chose surgery-oriented specialties were more likely to be highly extraverted and open, while the medical students who chose basic medicine were more likely to have higher neuroticism levels when compared with the other groups.
Specialty preferences of undergraduate medical students: What do they choose and why?	2024	Medical students	1921	Cross-sectional	Multiple traits	Preffered specialty	Participants who opted for clinical branches were more extraverted and more open-minded than those who chose nonclinical branches. Participants who opted for surgical branches were more extraverted, more conscientious, and had fewer negative emotions than their peers who chose medicine-related branches.
Factors associated with medical students' career choice in different specialties: a multiple cross-sectional questionnaire study at a German medical school	2024	Undergraduate students	683	Cross-sectional	Multiple traits	Preffered specialty	Our findings indicate, both in positive and negative aspects, that personality factors such as agreeableness, neuroticism, and openness influence the choice of specialty among medical students.

Personality Types of Medical Students in Terms of Their Choice of Medical Specialty: Cross-Sectional Study	2024	Medical students	2104	Cross-sectional	Multiple traits	Preffered specialty	There is a link between one's personality and their choice of medical specialty. Introverted, Observant, and Judging individuals showed a notable inclination toward Family Medicine, while Intuitive and Prospective traits were associated with an interest in Psychiatry, particularly in Child and Youth Psychiatry. Additionally, the Thinking trait was prevalent among students opting for surgical specialties such as Neurosurgery, Forensic Medicine, Plastic Surgery, and General Surgery, whereas the Feeling trait was more common among those choosing pediatric specialties.
Personality and interest in general practice: results from an online survey among medical students	2024	Medical students	628	Longitudinal	Multiple traits	Choice of general practice	Our study reveals that students' personality traits predict their interest in General Practice and their intention to choose it as a specialty.
The associations of temperament, character, anxiety, and specialty choice among sixth-year medical students: A cross-sectional study.	2020	Final-year medical students	151	Cross-sectional	Anxiety as a trait, temperament	Preferred specialty	Anxiety not predictive for choice of specialty. Harm avoidance, fear of uncertainty, shyness, fatigability, and sentimentality lower for surgery.
Personality traits predict a medical student	2022	Medical students	335	Cross-sectional	Psychopathy	Intention for surgery residency	Surgery choice: higher psychopathic traits:

preference to pursue a career in surgery							Machiavelism, self-centered impulsivity, social influence, and fearlessness.
Association between HEXACO personality traits and medical specialty preferences in Mexican medical students: a cross-sectional survey	2020	Medical students	292	Cross-sectional	Multiple traits	Place of residency	Surgery choice: higher extraversion and organization, less anxious and less emotionally attached to patients.
Holland's Theory Applied to Medical Specialty Choice	2004	Graduates of a combined BS/MD program	447	Longitudinal	Multiple traits	Preferred specialty (technique or patient-oriented)	No significant predictors found.
Work style preferences among medical specialties	2014	Medical students	355	Longitudinal	Multiple traits	Preferred specialty	Preference for internal medicine related to the desire to work with people rather than ideas or things.
Tolerance for Uncertainty, Personality Traits and Specialty Choice Among Medical Students	2021	Second-year and sixth-year and medical students.	362	Longitudinal	Tolerance for uncertainty	Preferred specialty	Surgery choice related to low tolerance for risk, ambiguity, and complexity. Neuroticism

							predicted surgery choice.
Personality and learning styles of final-year medical students and the impact of these variables on medical specialty choices	2014	Final-year medical students	170	Cross-sectional	Multiple traits	Preferred specialty	Not predictive.
The influence of gender and personality traits on the career planning of Swiss medical students	2003	Medical students	719	Cross-sectional	Multiple traits	Preferred specialty	Not predictive.
Factors influencing medical students' intentions to choose psychiatry as a career	1989	First-year medical students	99	Cross-sectional	Multiple traits	Intention of psychiatry specialization	Extraversion is predictive of psychiatry choice if there is a positive view of others towards psychiatry, positive students' view, and willingness to comply with others.
Female medical students: who might make the cut?	2012	Students in the second year of a six-year undergraduate medical program	580	Cross-sectional	Multiple traits	Preferred specialty	Surgery choice in women is predicted by lower agreeableness scores by women who were not interested in

							surgery. Also, they had higher neuroticism and agreeableness than males interested in surgery.
Modal personality and values of medical students	1986	Medical students	42	Cross-sectional	Multiple traits	Primary care and patient-oriented care specialty	Not predictive.
Studies in medical education: the role of cognitive and psychological characteristics as career choice correlates	1972	Medical students	230	Cross-sectional	Multiple traits	Preferred specialty	Gynecology was predicted by orientation to practical use, lower desire for abstract, and need for conventional obligations. Psychiatry was predicted by a lower orientation to practical use, an orientation to abstract, a higher appreciation of altruism and social relations, and greater need for self-analysis of behavior.
Impact of personality temperaments and characters	2014	Medical graduates	331	Longitudinal	Multiple traits	Preferred specialty	Patient-centered specialties were related to higher reward

on academic performance and specialty selection among a group of Egyptian medical graduates							dependence, persistence, and cooperativeness. Clinical pathology was related to the highest harm avoidance. Radiology had the lowest harm avoidance. Surgery had the highest self-directedness.
Personality profiles of rural longitudinal integrated clerkship students who choose family medicine	2015	Medical students	145	Cross-sectional	Multiple traits	Preferred specialty	Choice of family medicine had lower levels of Harm Avoidance, higher Reward Dependence in comparison to other matches.
Relationship between personality traits and choosing a medical specialty	2015	Medical students	358	Cross-sectional	Multiple traits	Preferred specialty	Surgery choice predicted in higher Exibition compared to general medicine. Specialized medicine predicted in higher Autonomy compared to general medicine. Medical students who chose general medicine scored significantly higher in Intraception and

							Succorance compared to students who chose surgical medicine.
Why medical students choose psychiatry - A 20 country cross-sectional survey	2014	Medical students	2198	Cross-sectional	Multiple traits	Preferred specialty	No significant predictors found.
Specialty choice, stress and personality: their relationships over time	1999	Medical students	314	Longitudinal	Depressive experiences	Preferred specialty	Psychiatrists had the highest self-criticism. Lowest self-criticism was in anaesthetics, surgeons, and general practitioners. Surgeons predicted by highest self efficacy.
New results relating the Myers-Briggs type indicator and medical specialty choice	1988	Medical students	521	Longitudinal	Multiple traits	Preferred specialty	The students choosing family medicine tended to be sensing, feeling, judging types; the students choosing obstetrics-gynecology tended to be sensing, thinking, judging types; and the students choosing psychiatry tended to

							be intuitive, feeling, perceiving types.
Personality and specialty interest in medical students	2008	Medical students	1076	Cross-sectional	Multiple traits	Preferred specialty	High scores on a measure of impulsive sensation seeking, and low scores on a measure of neuroticism anxiety are associated with preferences in surgical specialties at the beginning of medical school. Low scores on a measure of sociability are associated with preferences in hospital-based specialties at the beginning of medical school.
Assessment of personality type and medical specialty choice among medical students from Karachi; using Myers-Briggs type indicator (MBTI) tool	2017	Medical students		Cross-sectional	Multiple traits	Preferred specialty	Extroverted-Sensing-Feeling-Perceptive 11(2.8%), Extroverted-Sensing-Thinking-Judging 12(3%), Extroverted-Sensing-Feeling-Judging 5(1.3%), Introverted-Sensing-Feeling-Judging 6(1.5%),

							Introverted-Sensing-Thinking-Perceptive 7(1.8%) had a preference for surgery, medicine, gynecology, pediatrics, and cardiology, respectively.
Choice of medical specialty and personality traits measured with the EPQ-R(S) in medical students and specialist doctors	2019	Students and specialist doctors	48	Cross-sectional	Multiple traits	Preferred specialty	Personality not predictive.
Specialty choice preference of medical students according to personality traits by Five-Factor Model	2016	Medical students	110	Cross-sectional	Multiple traits	Preferred specialty	Medical students with more Agreeableness were more likely to prefer clinical medicine, and those with more Openness preferred medical departments.
Career specialty choice: A combined research-intervention project	2005	Medical students	161	Cross-sectional	Multiple traits	Preferred specialty	Medical students with more Agreeableness were more likely to prefer clinical medicine, and those with more Openness

							preferred medical departments.
Does surgery attract students who are more resistant to stress?	1984	Medical students	169	Cross-sectional	Multiple traits	Preferred specialty	Choice of family medicine had lower levels of Harm Avoidance, higher Reward Dependence in comparison to other matches.
Medical speciality choice: does personality matter?	2015	Physicians and medical students	468	Cross-sectional	Multiple traits	Preferred specialty	Overall, the results of the current study suggest that there is not a personality profile which is common to all doctors or to particular medical specialties.
Personality as a prognostic factor for specialty choice: A prospective study of 4 medical school classes	2008	Medical students	479	Cross-sectional	Multiple traits	Preferred specialty	There were differences among specialties for neuroticism, openness, and agreeableness, but not for extraversion or conscientiousness. Neuroticism scores for graduates entering internal medicine were higher than those for anesthesiology, and scores for

							graduates entering pediatrics or radiology were higher than those for anesthesiology and surgery. Openness scores for graduates entering psychiatry were higher than those for anesthesiology, dermatology, emergency medicine, family medicine, pediatrics, radiology, and surgery, and scores for graduates entering obstetrics-gynecology were higher than in anesthesiology, dermatology, radiology, and surgery. Conscientiousness scores for graduates entering radiology were lower than anesthesiology, dermatology, family medicine, internal medicine, internal medicine/pediatrics,

							and obstetrics-gynecology, and scores for graduates entering family medicine were higher than those for surgery.
Who picks psychiatry? Perceptions, preferences and personality of medical students	2011	Medical students	573	Cross-sectional	Multiple traits		This study found that openness to experience was strongly associated with favoring psychiatry as a specialty choice. With respect to pediatrics, there were Overall significant differences across the five personality factors between those who indicated ‘ highly likely, ‘ undecided ’ and ‘ not likely ’. Those who indicated ‘ highly likely ’ had significantly higher Extraversion and Agreeableness scores than those who indicated 'undecided.’
Ability of prospective	2007	Medical students	152	Cross-sectional	Multiple traits	Preferred specialty	Personality scores for medical students

assessment of personality profiles to predict the practice specialty of medical students							selecting psychiatry residencies showed greater degrees of neuroticism and openness. Students electing family practice also deviated from other specialties, showing a lower degree of neuroticism. Personality traits in prospective surgical residents did not differ from those of students choosing non-surgical residencies.
Personality types and specialist choices in medical students	2012	Medical students	590	Cross-sectional	Multiple traits	Preferred specialty	Low scores on sociability measures were associated with preferences in hospital-based specialties. High scores on a measure of impulsive sensation seeking and low scores on a measure of neuroticism-anxiety are associated with preferences in surgical specialties.
Importance of the big-five in	2020	Medical students	407	Cross-sectional	Multiple traits	Preferred specialty	We could not attribute the

the future medical specialty preference							differences in personality traits to specialty preference.
Personality factors and psychiatry specialty attraction	2001	Medical students	1484	Cross-sectional	Multiple traits	Preferred specialty	Students who are more emotionally immature, sensitive, and dependent, as well as more impulsive, unstable, and disorderly, tend to feel attracted to the specialty of Psychiatry.
Machiavellianism and medical career choices	1955	Medical students	238	Cross-sectional	Machiavellianism	Preferred specialty	There were no significant differences between scores of medical students selecting psychiatry as compared with scores of those choosing medicine, surgery, or other clinical specialties.
Personality traits predict a medical student preference to pursue a career in surgery	2017	Medical students	360	Cross-sectional	Psychopathy	Intention for surgery residency	Students aspiring to a surgical career exhibited higher PPI-R total score, self-centered impulsivity (SCI) factor score, Machiavellian egocentricity, social influence, and

							fearlessness content scale scores.
The association between personality traits and specialty preference among medical students in Jordan	2020	Medical students, graduates	1012	Cross-sectional	Multiple traits	Place of residency	Medical students and fresh medical graduates with more extraversion and conscientiousness preferred to be practicing clinicians and were more likely to pursue their careers in surgery-oriented specialties.
Intellectual, personality, and environmental factors in career specialty preferences	1971	Medical students	459	Cross-sectional	Multiple traits	Preferred specialty	No differences were found in values in dogmatism.
Are Surgeons Born or Made? A Comparison of Personality Traits and Learning Styles Between Surgical Trainees and Medical Students	2016	Medical students and surgical trainees	53	Cross-sectional	Multiple traits	Preferred specialty	The high conscientiousness, agreeableness, and openness to new experiences combined with relatively lower levels of neuroticism
The relative effectiveness of personality and academic measures in the	1982	Medical students	340	Cross-sectional	Multiple traits	Intention of psychiatry specialization	The best set of predictors to classify students into two groups, those interested in a

prediction of psychiatry or nonpsychiatry medical specialty preferences							psychiatry specialty, and those interested in other specialties, would be a combination of three POMS sub-scales and the Trait Anxiety measure from the STAI. In combination, these four measures could classify 84% of the students correctly.
A multivariate analysis of personality, values and expectations as correlates of career aspirations of final year medical students	2010	Medical students	179	Cross-sectional	Multiple traits	Preferred specialty	A multivariate analysis of personality, values, and expectations as correlates of career aspirations of final year medical students.
Personality influence in the predilection by the different specialties among medical students	2003	Medical students	1484	Cross-sectional	Multiple traits	Preferred specialty	Compared with the rest of the students, those who preferred the specialties of Pediatrics, Gynecology, and Sensorial Specialties (including Dermatology, Ophthalmology, Otorhinolaryngology

							, and Odontology) tended to be "effective" (A+), "trusted" (L-) and "practices" (M-). Those students who selected Psychiatry tended to be "sensible" (I+) and "imaginative" (M+). The students who preferred Orthopedic Surgery tended to be "Hard" (I-) and "Practices" (M-). The students who chose internal medicine or surgery have a personality profile that is similar to that of the general population.
Elección de una especialidad Médica-Quirúrgica: factores que intervienen = Election of a medical versus surgical speciality: The personality influence	2002	Medical students	1484	Cross-sectional	Multiple traits	Preffered specialty	The students who chose a "medical specialty" tend to be more "tender-minded, sensitive..." (I+), "trusting" (L-) and "shrewd, astute, calculating..." (N+) and who preferred a "surgical specialty" more "impulsive, happy-go-lucky,

							enthusiastic..." (F+) and "strong-minded, self-reliant..." (I-).
Personal characteristics of students choosing different types of medical careers	1964	Students at the end of their first year or internship training,		Longitudinal	Multiple traits	Preffered specialty	Students who choose general practice careers do not have as great a need to exercise leadership as their classmates who choose full-time specialty practice or part-time academic careers.
Psychiatry as a career choice among medical students: a cross-sectional study examining school-related and non-school factors	2018	Medical students	502	Cross-sectional	Multiple traits	Intention of psychiatry specialization	Among the self-rated personality traits, only agreeableness and neuroticism were found to be significant factors associated with choosing psychiatry as a career. Those unlikely to choose psychiatry scored significantly lower in these two personality traits.
Personality of medical students declaring surgical	2015	Medical students	234	Cross-sectional	Multiple traits	Intention for surgery residency	The personality constellation of future surgical adepts seems to be a coherent and

specialty choice in the context of prospective medical practice style]							strong predictor of the style of medical practice characteristic of surgery, characterized by a strong biotechnical orientation and, in the interpersonal layer, a predisposition to build paternalistic relationships with patients
Different but similar: personality traits of surgeons and internists-results of a cross-sectional observational study	2018	Medical students and physicians	1350	Cross-sectional	Multiple traits	Preferred specialty (technique or patient-oriented)	Between specialties, moderate differences exist: compared with medical doctors, on average, surgeons show lower levels of neuroticism, extraversion, and openness to experience, while there is no significant difference in agreeableness and conscientiousness
Myers-Briggs type and medical specialty choice:	2009	Medical students	3987	Longitudinal	Multiple traits	Preferred specialty	Those with a preference for introversion and feeling likely to choose primary care

a new look at an old question							specialties. Of those graduates who selected primary care, only feeling types chose Family Medicine more often than thinking types. Of those who selected nonprimary care, there was a higher proportion of males, extroverts, and thinking types in the surgical specialties.
Personality and values as predictors of medical specialty choice	2011	Medical students	244	Longitudina l	Multiple traits	Preferred specialty	Results indicated that first-year medical students who entered person-oriented specialties tend to be more sensitive than those who entered technique-oriented specialties. Medical students who entered person-oriented specialties also tended to display more warmth than those entering technique-oriented specialties. Additionally, those

							who elected to enter person-oriented specialties tended to be more rule-conscious and apprehensive compared to those who entered technique-oriented specialties. Conversely, first-year medical students electing to enter technique-oriented specialties tended to be more dominant, vigilant, and tense compared to those who entered person-oriented specialties.
Personality correlates of a career interest in psychiatry	1969	Medical students	403	Cross-sectional	Extraversion	Intention of psychiatry specialization	The students who favor abstract ideas are positive, and the students who prefer ideas with practical application are negative about a psychiatric career. Complexity, capacity to tolerate ambiguities also correlated with a

							positive psychiatric career attitude.
Young Doctors Aiming to Enter Different Specialties	1969	Medical student	120	Cross-sectional	Extraversion	Preferred specialty	Simple personality classification of specialists, therefore, is not to be expected.
Medical career choice and practice location: Early factors predicting course completion, career choice and practice location	2004	Medical student	229	Longitudinal	Multiple traits	Choice of general practice	Predictive factors for specialization were being more abstract in their thinking and more conscientious and rulebound.
The specialty choices of graduates from Brighton and Sussex Medica School: A longitudinal cohort study	2015	Medical students and graduates	105	Longitudinal	Multiple traits	Preferred spacialty and place of residency	Respondents who chose acute care were significantly more extroverted than those who chose medical specialties, psychiatry, and general practice.
The association between Myers-Briggs Type Indicator and Psychiatry as the specialty choice	2016	Medical students	835	Cross-sectional	Multiple traits	Place of residency	Aspiring psychiatrists showed preferences for Introversion over Extraversion, Intuition over Sensing, Judging

							over Perceiving, and no preference for Thinking vs Feeling.
Medical specialty choice and personality: I Initial results and predictions	1969	Medical students	190	Longitudinal	Multiple traits	Preferred specialty	Those students in "people-oriented" specialties and activities usually reflect personality needs relating more to affiliative desires and helping-out interests and were evaluated and observed to be more comfortable with people. Those students in specialties where involvement with others is more minimal (technique-oriented) had needs reflecting other orientations, such as being exhibitionistic or needing help themselves, and were assessed and observed to be less at ease with people.
Personality profiles and specialty choices of students from	1911	Medical students	199	Longitudinal	Multiple traits	Place of residency	The students who were entering internal medicine had few psychological

two medical school classes							features that distinguished them from their colleagues in other fields. They tended not to be anxiety-prone and were relatively low in intimacy motivation. Hospital-based specialties: They preferred and worked better in structured work settings that provided fixed guidelines. The surgeons also can be described as highly aggressive and tough-minded (mascu-linity-femininity), competitive, inde-pendent, and dominant (masculinity). Obstetricians: They are anxiety-prone (neuroticism), uncom-fortable around others (social anxi-ety), very concerned about appear-ances

							and making a good impression (public self-consciousness), and low in aggression, high in emotional vul-nerability (masculinity-femininity). The pediatricians had a number of traits in common with the obstetricians. They, too had an external locus of control, scored high on warmth and helpfulness (femininity), and were relatively low on the masculine traits of competitiveness, confidence, and independence. They were also the most extroverted or sociable of all the groups studied, unlike the stu- dents entering any other specialty. The students who entered psychia- try tended to be

							introverted and to have fewer social connections than their peers.
Authoritarianism and Machiavellianism among medical students.	1965	Medical students	2548	Cross-sectional	Multiple traits	Preferred specialty	In the present sample of medical students, it has been found that authoritarian students tend to select general prac-tice and reject internal medicine and psychiatry, while Machiavellian students tend to select psychiatry and reject the general practice.
Death anxiety, authoritarianism, and choice of specialty in medical students	1965	Medical students	114	Cross-sectional	Multiple traits	Preferred specialty	Future surgeons, internists, and pediatricians were each significantly more authoritarian than psychiatrists. Surgeons-to-be were significantly less anxious about death when compared to future internists, pediatricians, and psychiatrists. There is a tendency for

							future internists to have lower death anxiety than pediatricians. Potential psychiatrists are significantly lower in authoritarianism and higher in death anxiety when compared to the three other specialties combined.
Selection of medical specialties preferences versus choices	1977	Medical students	350	Cross-sectional	Intolerance uf uncertainty	Preferred specialty	It was hypothesized that if intolerance of ambiguity was a personality factor associated with specialty choice, students who preferred but did not choose the least structured specialty, psychiatry, would have less tolerance for ambiguity than students who chose psychiatry. Likewise, it was hypothesized that students who preferred but did not choose the most highly structured specialties of

							surgery and obstetrics-gynecology would have more tolerance for ambiguity than students who chose these two specialties.
