# Supplementary material for: Personality traits and medical specialty preference among medical students and graduates: a scoping review
Source: Croat Med J. 2025 Oct;66(5):321–33. doi: 10.3325/cmj.2025.66.321 (PMC12631570; doi:10.3325/cmj.2025.66.321)
Supplement: Supplementary Material 5 [file CroatMedJ_66_s005.pdf]

Supplemental Material 5

Table 1. Missing methodological elements of the Critical Appraisal Checklist for cross-sectional studies (N=47)

**Unfulfilled criteria of the Critical Appraisal Checklist**	**No.**	**References**
**Criteria for inclusion in the sample clearly defined**	24	(1–14,14–23)
**Was the exposure measured in a valid and reliable way?**	N/A	N/A
**Objective, standard criteria used for measurement of the condition**	8	(9,12–14,18,21,24,25)
**Confounding factors identified**	10	(4,5,13,14,14,16,22,24,26–28)
**Strategies to deal with confounding factors stated**	21	(1,2,4,5,8,9,12–14,16,18,20–24,26–31)
**Outcomes measured in a valid and reliable way**	4	(2,14,18,24)
**Appropriate statistical analysis used**	21	(1,2,4,9,12–14,16–18,20–27,31–33)

Table 2. Missing methodological elements of the Critical Appraisal Checklist for longitudinal studies (N=14)

Missing methodological elements of the Critical Appraisal Checklist	No.	References
Were the two groups similar and recruited from the same population?	/	/
Were the exposures measured similarly to assign people to both exposed and unexposed groups?	N/A	N/A
Was the exposure measured in a valid and reliable way?	N/A	N/A
Confounding factors identified	6	(34–39)
Strategies to deal with confounding factors stated	6	(34–39)
Strategies to address incomplete follow-up utilized unclear	3	(35,38,40)
Were the groups/participants free of the outcome at the start of the study (or at the moment of exposure)?	N/A	N/A
Were the outcomes measured in a valid and reliable way?	/	/
Was the follow up time reported and sufficient to be long enough for outcomes to occur?	/	/
Was follow up complete, and if not, were the reasons to loss to follow up described and explored?	/	/
Was appropriate statistical analysis used?	/	/
